# Effects of Dietary Highland Barley at Different Inclusion Levels on Normobaric Hypoxia Tolerance, Oxidative Stress, Energy Metabolism, and Gut Microbiota in Mice

**DOI:** 10.3390/nu18040659

**Published:** 2026-02-17

**Authors:** Liangxing Zhao, Luman Sang, Yan Liu, Baicheng Lai, Qingyu Zhao, Liang Zou, Qun Shen

**Affiliations:** 1 College of Food Science and Nutritional Engineering, China Agricultural University, Beijing 100083, China; zhaoliangxing23@163.com (L.Z.);; 2 National Grain Industry (Highland Barley Deep Processing) Technology Innovation Center, Beijing 100083, China; 3 National Grain and Oil Standards Research Verification and Testing Center, Beijing 100083, China; 4 Department of Physical Education, China Agricultural University, Beijing 100083, China; 5 Key Laboratory of Coarse Cereal Processing of Ministry of Agriculture and Rural Affairs, Chengdu University, Chengdu 610106, China; 6 Chengdu Agricultural College, Chengdu 611130, China

**Keywords:** highland barley, hypoxia tolerance, dose–response, oxidative stress, gut microbiota

## Abstract

Background: Normobaric acute hypoxia models are widely applied to assess tolerance to acute hypoxic stress. Highland barley is a cereal crop originating from and traditionally cultivated in high-altitude regions; however, the dose–response relationship underlying its effects on hypoxia tolerance remains unclear. Methods: Male ICR mice were randomly allocated to five groups (*n* = 8 per group) and fed an AIN-93M basal diet or experimental diets supplemented with 20%, 40%, 60%, or 80% highland barley for 13 weeks. Hypoxia survival time was evaluated using a normobaric asphyxial hypoxia model, in which oxygen is progressively depleted in a sealed chamber by continuous respiration with carbon dioxide absorbed by soda lime. Hematological parameters, indices of oxidative stress and energy metabolism, and gut microbiota composition were also assessed. Results: Compared with the control group, dietary supplementation with 20% highland barley was associated with a longer hypoxia survival time (mean difference: 9.49 min; 95% CI: −2.05 to 21.02), whereas the 80% group exhibited the shortest survival time (approximately 40.6 min). In the 20% group, red blood cell count and hemoglobin concentration increased by 41.6% and 42.1%, respectively. ATP content and superoxide dismutase activity in brain tissue increased by 33.2% and 28.4%, respectively, with similar trends observed in heart tissue. In addition, gut microbiota α-diversity was increased in the 20% highland barley group, and distinct separation of microbial community structures was observed among groups receiving different supplementation levels. Conclusions: Overall, the data suggest that moderate dietary supplementation with highland barley (20%) is associated with a favorable physiological and microbiota profile under normobaric asphyxial hypoxic challenge, suggesting the presence of a potentially effective intake range for highland barley-based nutritional intervention.

## 1. Introduction

Normobaric acute hypoxia is a stress condition characterized by a transient restriction of oxygen availability under normal atmospheric pressure and is commonly associated with reduced exercise tolerance, impaired energy metabolism efficiency, and compromised overall physiological function [1,2]. Because of its widespread occurrence, controllable induction, and clearly defined physiological responses, this form of hypoxia has attracted considerable attention in the fields of physiology, exercise science, and nutritional intervention research [3]. Under acute hypoxic conditions, the capacity of the organism to sustain vital activities and preserve the function of key organs—defined as hypoxia tolerance—is considered a critical functional indicator of systemic physiological homeostasis and stress adaptation [4]. Current evidence suggests that hypoxia tolerance is not determined by a single factor but instead reflects the integrated regulation of multiple physiological processes, including oxygen transport capacity, energy metabolism efficiency, oxidative stress regulation, and alterations in gut microbiota composition [4,5,6].

In addition to pharmacological interventions such as dexamethasone and acetazolamide [7], growing interest has been directed toward the potential of common dietary components, including quinoa and wheat, to modulate hypoxia tolerance. For example, ethanol extracts of quinoa have been shown to alleviate hypoxia-induced oxidative stress by reducing H_2_O_2_ and malondialdehyde (MDA) levels while enhancing superoxide dismutase (SOD) and total antioxidant capacity (T-AOC) activities. These effects were accompanied by the maintenance of ATP levels and suppression of abnormal lactate dehydrogenase (LDH) elevation, thereby contributing to the preservation of energy metabolism homeostasis and improvement of overall tolerance under acute hypoxic conditions [8]. In addition, peptides derived from wheat germ have been reported to prolong survival time under normobaric acute hypoxia, with associated reductions in oxidative stress (H_2_O_2_ and MDA), enhancement of antioxidant defense capacity (SOD and total antioxidant capacity), and improvement of brain energy metabolism, as evidenced by increased ATP levels and decreased LDH activity [9]. Compared with pharmacological approaches, dietary interventions offer advantages including a favorable safety profile and feasibility for long-term application and are therefore considered a promising strategy for the modulation of hypoxia tolerance [10]. However, systematic investigations of dietary interventions in the regulation of tolerance to acute asphyxial hypoxia remain limited, particularly with respect to dose–response relationships.

Highland barley (*Hordeum vulgare* L.) is a cereal crop predominantly cultivated in high-altitude regions and is rich in dietary fiber, β-glucan, plant proteins, and a range of bioactive components. In recent years, it has attracted considerable interest in the fields of nutrition and health [11]. Previous studies have shown that dietary supplementation with highland barley can improve metabolic status, partly through the elevation of ATP levels [12]. In addition, highland barley intervention has been reported to enhance total antioxidant capacity (T-AOC), thereby strengthening antioxidant defense and alleviating oxidative stress [13]. Furthermore, consumption of highland barley has been associated with increased abundance of beneficial gut microbiota, including *Bifidobacterium* [14] and *Lachnospiraceae_NK4A136_group* [15], as well as modulation of overall gut microbial structure. Together, these findings provide indirect evidence supporting a potential role for highland barley in the regulation of hypoxia tolerance. However, evidence linking gut microbial alterations to acute hypoxia tolerance remains largely correlational, and the physiological relevance of these associations warrants further substantiation through quantitative association analyses. Moreover, evidence from dietary intervention studies indicates that dose–response relationships are often non-linear, and higher intake levels do not necessarily translate into greater physiological benefits [16]. Accordingly, we adopted a relatively wide range of highland barley inclusion levels (20–80%) to capture both potentially effective and potentially excessive intakes, thereby enabling identification of an intake range with practical nutritional relevance.

Based on the above background, male ICR mice were used as an experimental model to evaluate the effects of dietary supplementation with 20%, 40%, 60%, or 80% highland barley, with an AIN-93M basal diet serving as the control. The impact of different levels of highland barley intake on normobaric acute hypoxia tolerance was systematically assessed. Hypoxia survival time was determined using a normobaric asphyxial hypoxia model. In parallel, body weight and food intake, peripheral red blood cell count and hemoglobin concentration, as well as indices related to oxidative stress and energy metabolism in brain and heart tissues were analyzed to comprehensively characterize the physiological responses of key high–oxygen-demand organs to highland barley dietary intervention. In addition, gut microbiota composition was analyzed using 16S rRNA gene sequencing to explore its potential association with hypoxia tolerance. This study aimed to elucidate the effects of highland barley dietary intervention on hypoxia tolerance and its dose-dependent characteristics, thereby providing experimental evidence to support the rational dietary application of highland barley as a functional cereal and offering new insights into nutritional strategies for improving hypoxia tolerance.

## 2. Materials and Methods

### 2.1. Materials and Equipment

Raw highland barley powder (cultivar “Zangqing No. 3”) was provided by Shanxi Dongfangliang Life Science and Technology Co., Ltd. (Taiyuan, China). A standard purified maintenance diet (AIN-93M) was purchased from Changzhou Shuyi Shuer Biotechnology Co., Ltd. (Changzhou, China).

A GL-20G-II refrigerated centrifuge was obtained from Shanghai Anting Scientific Instrument Factory (Shanghai, China). A Multiskan™ GO microplate reader was purchased from Thermo Fisher Scientific (Waltham, MA, USA). An ultra-high-performance liquid chromatography (UHPLC) system (Dionex, Sunnyvale, CA, USA) was coupled to a Q Exactive Plus high-resolution tandem mass spectrometer (Thermo Fisher Scientific, Waltham, MA, USA). A PCR thermocycler was obtained from Applied Biosystems (ABI, Foster City, CA, USA), and sequencing was performed using an Illumina sequencing platform (Illumina, San Diego, CA, USA).

### 2.2. Methods

#### 2.2.1. Determination of Basic Nutritional Composition of Highland Barley

The nutritional composition of highland barley was determined according to the method described by Zhao et al. [12]. The corresponding nutritional data have been reported in our previous study and were used as the basis for the formulation of experimental diets in the present study [12].

#### 2.2.2. Animal Experimental Design

Male ICR mice (4 weeks of age, body weight 26–28 g) were obtained from Beijing Vital River Laboratory Animal Technology Co., Ltd. (Beijing, China; license number: SCXK (J) 2021-0006). All animals were specific pathogen–free (SPF) and housed under controlled environmental conditions (22 ± 1 °C, 55 ± 10% relative humidity, and a 12 h light/dark cycle), with ad libitum access to food and water. Body weight and food intake were recorded weekly throughout the experimental period.

All animal experimental procedures were conducted in accordance with the guidelines of the National Research Council and were approved by the Animal Ethics Committee of China Agricultural University (approval number: AW12011202-4-1; approval date: 25 October 2021).

After a one-week acclimatization period, mice were randomly allocated to five groups (*n* = 8 per group): a normal control group (NC, AIN-93M diet), a 20% highland barley group (HB20), a 40% highland barley group (HB40), a 60% highland barley group (HB60), and an 80% highland barley group (HB80). The sample size (*n* = 8 per group) was determined based on group sizes commonly used in previous normobaric hypoxia and related physiological intervention studies [8,12,17].

Experimental diets were custom-formulated by Changzhou Shu Yi Shu Er Biotechnology Co., Ltd. (Changzhou, China). The proportions of energy derived from protein, fat, and carbohydrates were identical across all diets (Table S1), and detailed diet compositions are provided in Table S2.

After 13 weeks of dietary intervention, all mice were subjected to a normobaric hypoxia survival test.

#### 2.2.3. Normobaric Hypoxia Survival Test in Mice

The normobaric hypoxia survival test was performed according to Wang et al. [17], with minor modifications. Briefly, mice from each group were individually placed into 250 mL ground-glass bottles (one mouse per bottle), and 10 g of soda lime was added to absorb exhaled carbon dioxide. Each bottle was connected via a rubber stopper and glass tubing to a graduated cylinder filled with water, with an additional buffer bottle positioned between the ground-glass bottle and the graduated cylinder to prevent water backflow. All connections and bottle openings were sealed with petroleum jelly, and the glass tubing was inserted below the water surface.

All tests were conducted under standardized and controlled environmental conditions (constant room temperature, quiet setting, and minimal variation in lighting and personnel movement) to reduce external disturbances. Mice from different dietary groups were tested in a prespecified alternating order to minimize potential order-related bias. The experiment was initiated immediately after sealing the system, and survival time was recorded under sealed normobaric conditions as oxygen was progressively depleted. The survival endpoint was defined using a prespecified, operational criterion: persistent absence of visible thoracic respiratory movement for ≥30 s. Endpoint determination was independently observed by two investigators at the same time point and recorded only after concordant confirmation. The personnel responsible for time recording were not involved in animal group allocation or diet preparation.

Immediately after the survival endpoint was reached, samples were collected for downstream biochemical analyses (hematological parameters, oxidative stress indices, and energy metabolism indices). Because these measurements were obtained under the same terminal hypoxic challenge conditions for all groups, they were used to compare group-level physiological responses to an identical acute hypoxic stress rather than to represent the resting baseline metabolic state after the 13-week dietary intervention. This endpoint sampling strategy is consistent with commonly used designs in normobaric asphyxial hypoxia studies [9].

#### 2.2.4. Determination of Hematological Parameters

Immediately after completion of the normobaric hypoxia survival test, blood samples were collected by cardiac puncture using sterile disposable syringes. Whole blood samples were transferred into tubes containing K_2_-EDTA anticoagulant, gently mixed, and analyzed immediately. Red blood cell count (RBC) and hemoglobin concentration (HGB) were measured using an automated animal hematology analyzer (Mindray DC-5180, Mindray Bio-Medical Electronics Co., Ltd., Shenzhen, China). Instrument settings were calibrated according to the mouse species prior to analysis.

#### 2.2.5. Determination of Oxidative Stress Indices in Brain and Cardiac Tissues

Immediately after completion of the normobaric hypoxia survival test, mice were sacrificed, and brain and heart tissues were rapidly excised, rinsed with ice-cold physiological saline to remove residual blood, blotted dry, and processed on ice throughout the procedure. Tissue samples were weighed and homogenized in pre-chilled physiological saline at a ratio of 1:9 (*w*/*v*) using a mechanical homogenizer under cold conditions. The homogenates were centrifuged at 3000× *g* for 10 min at 4 °C, and the supernatants were collected for subsequent analyses. Levels of hydrogen peroxide (H_2_O_2_), malondialdehyde (MDA), total antioxidant capacity (T-AOC, expressed as Trolox equivalents), superoxide dismutase (SOD), glutathione peroxidase (GSH-Px), and catalase (CAT) in brain and heart tissues were determined using commercial assay kits purchased from Nanjing Jiancheng Bioengineering Institute (Nanjing, China), including H_2_O_2_ (Cat. No. A064-1-1), MDA (Cat. No. A003-1-2), T-AOC (Cat. No. A015-2-1), GSH-Px (Cat. No. A005-1-2), SOD (Cat. No. A001-1-2), and CAT (Cat. No. A007-1-1), following the manufacturer’s instructions. According to the information provided in the kit manuals, the reported intra-assay and inter-assay coefficients of variation for these assays were generally within 10%. Total protein content in each sample was determined using a bicinchoninic acid (BCA) protein assay kit (Solarbio, Beijing, China). All oxidative stress parameters were normalized to protein content and expressed per milligram of protein.

#### 2.2.6. Determination of Energy Metabolism Indices in Brain and Cardiac Tissues

Brain and heart tissues were collected immediately after the hypoxia survival test and processed on ice as described above. Samples were homogenized in pre-chilled physiological saline at a ratio of 1:9 (*w*/*v*) and centrifuged at 3000× *g* for 10 min at 4 °C to obtain supernatants for analysis. Adenosine triphosphate (ATP) content and lactate dehydrogenase (LDH) activity in brain and heart tissues were measured using commercial assay kits from Nanjing Jiancheng Bioengineering Institute (Nanjing, China), including ATP (Cat. No. A070-2-2) and LDH (Cat. No. A020-2-2), according to the manufacturer’s protocols. According to the information provided in the kit manuals, the reported intra-assay and inter-assay coefficients of variation for these assays were generally within 10%. Total protein concentration was determined using a BCA protein assay kit (Solarbio, Beijing, China), and ATP and LDH values were normalized to protein content and expressed per milligram of protein.

#### 2.2.7. 16S rRNA Gene Sequencing and Gut Microbiota Analysis

Gut microbiota analysis was conducted following standard protocols with minor modifications [12,18]. Cecal contents samples were collected aseptically, immediately frozen in liquid nitrogen, and stored at −80 °C until analysis. Microbial genomic DNA was extracted using the E.Z.N.A.^®^ Soil DNA Kit (Omega Bio-Tek, Norcross, GA, USA) according to the manufacturer’s instructions. DNA quality was assessed by 1% agarose gel electrophoresis, and DNA concentration and purity were determined using a NanoDrop 2000 spectrophotometer (Thermo Scientific, Wilmington, DE, USA). The V3–V4 hypervariable region of the bacterial 16S rRNA gene was amplified using primers 338F (5′-ACTCCTACGGAGGCAGCAG-3′) and 806R (5′-GGACTACHVGGTWTCTAAT-3′). Purified amplicons were pooled in equimolar concentrations and sequenced on an Illumina MiSeq platform (PE300). Raw paired-end reads were demultiplexed, quality-filtered, and merged using FLASH (v1.2.7). High-quality sequences were clustered into operational taxonomic units (OTUs) at 97% sequence similarity using UPARSE (v7.1), with chimeric sequences removed during the clustering process. Representative OTU sequences were taxonomically assigned using the RDP classifier (confidence threshold = 0.7) against the SILVA rRNA database (version 138).

After quality control, high-quality sequences were obtained from 30 cecal contents samples, with sequencing depths ranging from 32,704 to 149,838 reads per sample (median: 49,267; mean: 52,948; interquartile range: 3752 reads). These 30 samples were those that met DNA extraction and sequencing quality-control criteria for microbiota analysis; all other physiological and biochemical endpoints were evaluated in the full cohort (*n* = 40). To minimize bias introduced by uneven sequencing depth, all samples were rarefied to 32,000 reads per sample prior to downstream alpha- and beta-diversity analyses.

Alpha-diversity indices were calculated based on the rarefied OTU table. Beta-diversity was assessed using Bray–Curtis distances and visualized by principal coordinates analysis (PCoA). Statistical differences in community structure among groups were evaluated using non-parametric tests as appropriate. Differentially abundant taxa were identified using LEfSe analysis with a linear discriminant analysis (LDA) score threshold of 3.0.

#### 2.2.8. Data Processing and Statistical Analysis

Data are presented as mean ± SEM. Statistical analyses were performed using SPSS software (version 22.0; IBM Corporation, Chicago, IL, USA), and figures were generated using GraphPad Prism (version 9.0.1). Prior to parametric analyses, data normality was assessed using the Shapiro–Wilk test, and homogeneity of variances was evaluated using Levene’s test. When these assumptions were met, differences among multiple groups were analyzed by one-way analysis of variance (ANOVA). If the omnibus ANOVA indicated a significant group effect, Duncan’s multiple range test was applied for post hoc pairwise comparisons to characterize differences among dietary inclusion levels. This approach was used to characterize group separation across multiple dietary inclusion levels [19,20]. A two-sided *p* value < 0.05 was considered statistically significant. To enhance transparency and interpretability, effect sizes for key outcomes were additionally reported in the Results section as between-group mean differences with corresponding 95% confidence intervals (CIs), particularly for the primary endpoint (hypoxia survival time) and other major physiological indicators.

## 3. Results

### 3.1. Effects of Highland Barley Dietary Intervention on Body Weight, Food Intake, and Hypoxia Tolerance in Mice

**Figure 1:**
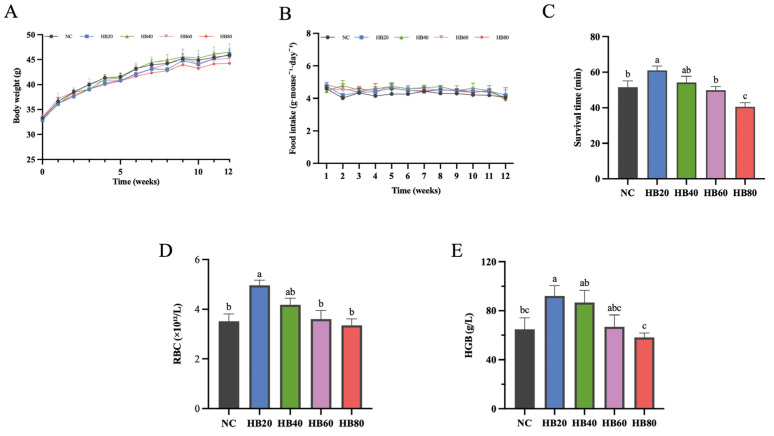
Effects of dietary highland barley intervention on tolerance to normobaric hypoxia, body weight, food intake, and hematological parameters in mice. (**A**) body weight change; (**B**) food intake; (**C**) survival time under normobaric hypoxia; (**D**) red blood cell count (RBC); (**E**) hemoglobin (HGB). Note: Data are presented as mean ± SEM (*n* = 8 mice per group). Group differences were analyzed by one-way ANOVA followed by Duncan’s multiple range test when the omnibus test was significant. Bars sharing the same letter (a–c) are not significantly different, whereas bars with different letters indicate statistically significant differences (*p* < 0.05).

During the entire intervention period, body weight in all groups gradually increased over time, and similar trends in body weight changes were observed among the different dietary groups, with no significant differences detected between groups (Figure 1A). Meanwhile, the average daily food intake of mice in all groups remained relatively stable throughout the experiment, and no significant differences were observed among the dietary groups (Figure 1B), indicating that dietary supplementation with different proportions of highland barley did not markedly affect overall growth status or feeding behavior in mice.

Under the normobaric acute asphyxial hypoxia model, hypoxia survival time (min) for each group is shown in Figure 1C. Compared with the basal diet control group (NC), mice in the HB20 group showed a numerically longer hypoxia survival time (61.04 ± 2.39 min vs. 51.56 ± 3.52 min), corresponding to an estimated mean difference of 9.49 min (95% CI: −2.05 to 21.02). The survival time of mice in the HB40 group was 54.18 ± 3.55 min, which was slightly higher than that of the NC group, although the difference did not reach statistical significance. With further increases in highland barley supplementation, hypoxia survival time in the HB60 group was 49.87 ± 2.08 min, comparable to that of the NC group. In contrast, the HB80 group exhibited the shortest hypoxia survival time (40.56 ± 2.29 min). Compared with the HB20 group, survival time was significantly reduced by 20.49 min (95% CI: 8.95–32.02 min, *p* < 0.05). Relative to NC, the estimated difference for HB80 was −11.00 min (95% CI: −22.53 to 0.53).

Overall, highland barley supplementation altered hypoxia tolerance without affecting body weight or food intake, with the longest survival time observed in the HB20 group.

### 3.2. Effects of Highland Barley Dietary Intervention on Hematological Parameters

Highland barley dietary intervention significantly affected hematological parameters in mice (Figure 1D,E). Compared with the basal diet control group (NC), red blood cell count (RBC, ×10^12^/L) was significantly increased in the HB20 group, rising from 3.51 ± 0.29 to 4.97 ± 0.20. The estimated mean difference between HB20 and NC was 1.46 ×10^12^/L (95% CI: 0.33–2.59 ×10^12^/L). The HB40 group exhibited an intermediate RBC level (4.18 ± 0.26), whereas no significant differences were observed between the HB60 group (3.60 ± 0.35), the HB80 group (3.35 ± 0.26), and the NC group (Figure 1D).

Hemoglobin concentration (HGB, g/L) displayed a trend consistent with the changes observed in RBC (Figure 1E). HGB levels were higher in the HB20 group than in the NC group (92.00 ± 8.53 vs. 64.75 ± 9.50), corresponding to an estimated mean difference of 27.25 (95% CI: −7.47 to 61.97). The HB40 group showed an increased HGB level (86.75 ± 9.77), although the difference did not reach statistical significance. In contrast, the HB80 group exhibited a reduced HGB level of 58.25 ± 3.56, which was significantly lower than that in the HB20 group (*p* < 0.05). No significant difference was observed between the HB60 group (66.88 ± 9.67) and the NC group.

Taken together, these results demonstrate that highland barley dietary intervention modulated hematological parameters in mice, with a 20% inclusion level exerting the most pronounced effects on RBC and HGB. Further increases in highland barley inclusion were associated with a gradual decline in these parameters.

### 3.3. Effects of Highland Barley Dietary Intervention on Oxidative Stress Status in Brain and Heart Tissues

**Figure 2:**
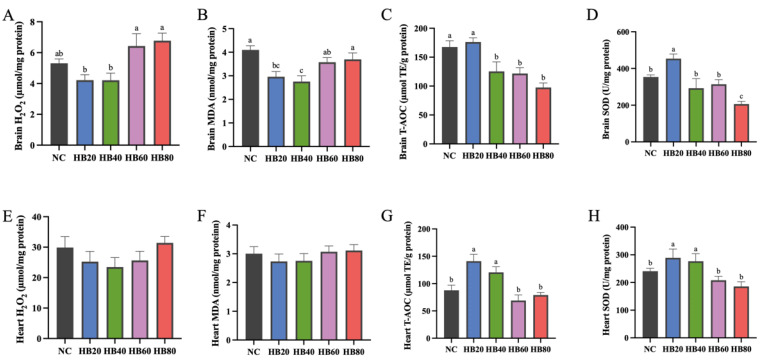
Effects of dietary highland barley intervention on oxidative stress status and antioxidant capacity in brain and heart tissues of mice under normobaric hypoxia. (**A**) H_2_O_2_ content in brain tissue; (**B**) malondialdehyde (MDA) content in brain tissue; (**C**) total antioxidant capacity (T-AOC) in brain tissue; (**D**) superoxide dismutase (SOD) activity in brain tissue; (**E**) H_2_O_2_ content in heart tissue; (**F**) malondialdehyde (MDA) content in heart tissue; (**G**) total antioxidant capacity (T-AOC) in heart tissue; (**H**) superoxide dismutase (SOD) activity in heart tissue. Note: Data are presented as mean ± SEM (*n* = 8 mice per group). Group differences were analyzed by one-way ANOVA followed by Duncan’s multiple range test when the omnibus test was significant. Bars sharing the same letter (a–c) are not significantly different, whereas bars with different letters indicate statistically significant differences (*p* < 0.05).

To evaluate the effects of highland barley dietary intervention on oxidative stress status under hypoxic conditions, oxidative stress–related parameters were measured in brain and heart tissues, and the results are shown in Figure 2A–H.

In brain tissue, hydrogen peroxide (H_2_O_2_, μmol/mg protein) levels differed among dietary groups. Compared with the NC group (5.31 ± 0.28), H_2_O_2_ levels in the HB20 (4.22 ± 0.35) and HB40 (4.21 ± 0.46) groups showed a decreasing trend, although the differences did not reach statistical significance (*p* > 0.05). In contrast, H_2_O_2_ levels in the HB80 group were significantly higher than those in the HB20 and HB40 groups (*p* < 0.05) but were not significantly different from those in the NC group. Malondialdehyde (MDA, nmol/mg protein) levels in brain tissue were significantly reduced in the HB20 group compared with the NC group, decreasing from 4.10 ± 0.18 to 2.96 ± 0.22 (*p* < 0.05), whereas the HB80 group showed an MDA level of 3.70 ± 0.28 that did not differ significantly from that of the NC group (Figure 2B).

Antioxidant defense parameters in brain tissue exhibited a clear dose-dependent pattern. Total antioxidant capacity (T-AOC, μmol TE/g protein) and superoxide dismutase (SOD, U/mg protein) activity both reached their highest levels in the HB20 group. Compared with the NC group, T-AOC increased from 167.60 ± 10.82 to 176.24 ± 7.12, and SOD activity increased from 353.21 ± 11.52 to 453.68 ± 25.16 (*p* < 0.05). With further increases in highland barley supplementation, both parameters gradually declined, and values in the HB80 group were significantly lower than those in the HB20 group (*p* < 0.05) (Figure 2C,D).

In heart tissue, H_2_O_2_ and MDA levels showed relatively small variations and did not differ significantly among groups (Figure 2E,F). However, T-AOC and SOD activity were significantly elevated in the HB20 and HB40 groups compared with the NC group (*p* < 0.05), whereas both parameters were significantly reduced in the HB60 and HB80 groups (*p* < 0.05) (Figure 2G,H).

Taken together, these results demonstrate that highland barley dietary intervention modulated oxidative stress status in both brain and heart tissues. H_2_O_2_ and MDA levels exhibited a U-shaped response across increasing supplementation levels, whereas T-AOC and SOD activity followed an inverted U-shaped pattern, with the most favorable antioxidant responses observed at a 20% inclusion level.

### 3.4. Effects of Highland Barley Dietary Intervention on Energy Metabolism in Brain and Heart Tissues

**Figure 3:**
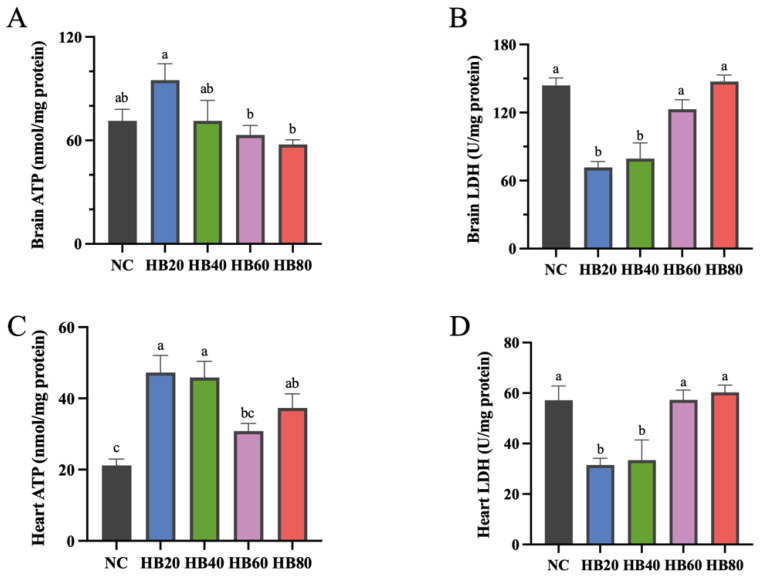
Effects of dietary highland barley intervention on energy metabolism–related parameters in brain and heart tissues of mice under normobaric hypoxia. (**A**) ATP content in brain tissue; (**B**) lactate dehydrogenase (LDH) activity in brain tissue; (**C**) ATP content in heart tissue; (**D**) lactate dehydrogenase (LDH) activity in heart tissue. Note: Data are presented as mean ± SEM (*n* = 8 mice per group). Group differences were analyzed by one-way ANOVA followed by Duncan’s multiple range test when the omnibus test was significant. Bars sharing the same letter (a–c) are not significantly different, whereas bars with different letters indicate statistically significant differences (*p* < 0.05).

To evaluate the effects of highland barley dietary intervention on energy metabolism under hypoxic conditions, ATP content and lactate dehydrogenase (LDH) activity were measured in brain and heart tissues, and the results are shown in Figure 3A–D.

In brain tissue, ATP content differed among dietary groups (Figure 3A). Compared with the basal diet control group (NC, 71.33 ± 6.76), ATP content increased to 94.99 ± 9.48 in the HB20 group, representing the highest level among all groups, although the difference did not reach statistical significance. ATP levels in the HB40 group were comparable to those in the NC group. With further increases in highland barley supplementation, ATP content in the HB60 and HB80 groups gradually declined, with the HB80 group showing a significantly lower level than the HB20 group (*p* < 0.05). In parallel, LDH activity in brain tissue differed significantly among dietary groups (Figure 3B). LDH activity was significantly lower in the HB20 and HB40 groups than in the NC group (143.99 ± 6.54), whereas it increased markedly in the high-supplementation groups. The HB80 group exhibited a significantly higher LDH activity than the HB20 group (*p* < 0.05).

In heart tissue, ATP content also displayed a dose-related pattern (Figure 3C). Compared with the NC group (21.19 ± 1.79), ATP levels were higher in the HB20 (47.26 ± 4.80) and HB40 (45.89 ± 4.54) groups, corresponding to estimated mean differences of 26.08 (95% CI: 11.17–40.98) and 24.71 (95% CI: 9.80–39.62), respectively. With further increases in highland barley inclusion, ATP levels declined relative to the moderate-intake groups. In the HB60 group, the difference from NC was smaller and not statistically significant (mean difference: 9.63, 95% CI: −5.27 to 24.54), whereas the HB80 group showed a modest increase compared with NC (mean difference: 16.12, 95% CI: 1.21–31.03). Correspondingly, LDH activity in heart tissue was significantly lower in the HB20 and HB40 groups than in the NC group, whereas it increased in the high-supplementation groups to levels comparable to those of the NC group and was significantly higher than that in the HB20 group (*p* < 0.05) (Figure 3D).

Taken together, these results demonstrate that highland barley dietary intervention exerted pronounced dose-dependent effects on energy metabolism in brain and heart tissues. ATP content followed an inverted U-shaped response pattern across increasing supplementation levels, whereas LDH activity exhibited a U-shaped response, with more favorable energy metabolic profiles observed at moderate supplementation levels.

### 3.5. Effects of Highland Barley Dietary Intervention on Gut Microbiota Composition

**Figure 4:**
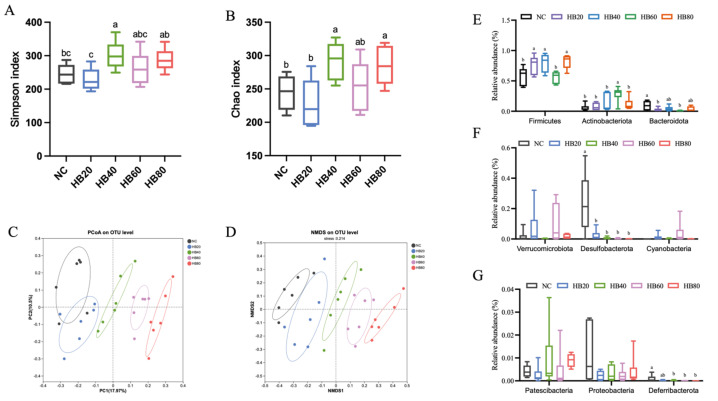
Effects of dietary highland barley intervention on gut microbiota diversity and community structure in mice under normobaric hypoxia. (**A**) Simpson index; (**B**) Chao index; (**C**) principal coordinates analysis (PCoA) at the OTU level; (**D**) non-metric multidimensional scaling (NMDS) at the OTU level; (**E**) Firmicutes, Actinobacteriota, and Bacteroidota; (**F**) Verrucomicrobiota, Desulfobacterota, and Cyanobacteria; (**G**) Patescibacteria, Proteobacteria, and Deferribacterota. Note: Data are presented as mean ± SEM (*n* = 6 mice per group). Group differences were analyzed by one-way ANOVA followed by Duncan’s multiple range test when the omnibus test was significant. Bars sharing the same letter (a–c) are not significantly different, whereas bars with different letters indicate statistically significant differences (*p* < 0.05).

To evaluate the effects of highland barley dietary intervention on gut microbiota composition under normobaric hypoxic conditions, 16S rRNA gene sequencing was performed on cecal contents from mice in each group. Gut microbial diversity and community structure were subsequently analyzed, and the results are presented in Figure 4.

Significant differences in α-diversity were observed among dietary groups (Figure 4A,B). Compared with the basal diet control group (NC), both Simpson and Chao indices were significantly increased in the HB20 and HB40 groups (*p* < 0.05), indicating enhanced gut microbial richness and evenness at moderate supplementation levels. In contrast, no further increases in α-diversity were observed in the high-supplementation groups (HB60 and HB80), and the Simpson index showed a decreasing trend.

β-diversity analysis further demonstrated clear separation of overall gut microbial community structures among dietary groups (Figure 4C,D). PCoA and NMDS analyses based on OTU-level data revealed distinct clustering of samples in two-dimensional space, suggesting that highland barley dietary intervention markedly reshaped gut microbial community structure, with evident differences among supplementation levels.

At the community composition level, relative abundance analysis at the phylum level showed that the proportions of major bacterial phyla varied among dietary groups (Figure S1A). Firmicutes, Actinobacteriota, and Bacteroidota were the dominant phyla across all groups, and their relative abundances differed significantly among groups with different levels of highland barley supplementation (*p* < 0.05). In addition, several low-abundance phyla, including Verrucomicrobiota, Desulfobacterota, and Proteobacteria, also exhibited differential changes across dietary groups (Figure 4F,G).

**Figure 5:**
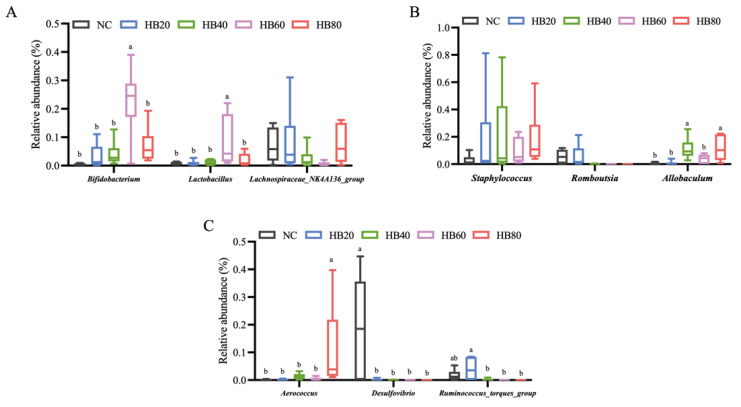
Differential gut microbial taxa under dietary highland barley intervention in mice exposed to normobaric hypoxia. (**A**) Bifidobacterium, Lactobacillus, and Lachnospiraceae_NK4A136_group; (**B**) Staphylococcus, Romboutsia, and Allobaculum; (**C**) Aerococcus, Desulfovibrio, and Ruminococcus_torques_group; Note: Data are presented as mean ± SEM (*n* = 6 mice per group). Group differences were analyzed by one-way ANOVA followed by Duncan’s multiple range test when the omnibus test was significant. Bars sharing the same letter (a,b) are not significantly different, whereas bars with different letters indicate statistically significant differences (*p* < 0.05).

Genus-level analysis further demonstrated pronounced differences in gut microbiota composition among the different highland barley dietary groups (Figure S1A). To further delineate key genus-level changes associated with highland barley dietary intervention, representative genera with relatively high abundance or significant differences were quantitatively compared (Figure 5A–C).

As shown in Figure 5A, *Bifidobacterium*, *Lactobacillus*, and *Lachnospiraceae_NK4A136_group* exhibited distinct distribution patterns across dietary groups. The relative abundance of *Bifidobacterium* was significantly increased in the HB60 group, whereas moderate levels were observed in the HB20 and HB40 groups. *Lactobacillus* was significantly enriched in the HB60 group compared with the other groups (*p* < 0.05). In contrast, *Lachnospiraceae_NK4A136_group* showed relatively higher abundance in the HB20 and HB80 groups, displaying a nonlinear distribution pattern across increasing highland barley inclusion levels. In another set of representative genera (Figure 5B), *Staphylococcus* exhibited higher relative abundance in the HB20 and HB40 groups than in the control group, but decreased in the HB60 group, although these differences did not reach statistical significance. *Romboutsia* showed relatively higher abundance in the HB20 group, followed by a gradual decline with increasing highland barley supplementation. *Allobaculum* displayed significantly higher relative abundance in the HB40 and HB80 groups compared with the low-supplementation groups (*p* < 0.05). As shown in Figure 5C, *Aerococcus* exhibited significantly higher relative abundance in the HB80 group, while remaining at low levels in the other groups (*p* < 0.05). *Desulfovibrio* showed relatively high abundance in the control group and exhibited a significant overall decrease across the highland barley intervention groups (*p* < 0.05). In addition, *Ruminococcus_torques_group* was significantly enriched in the HB20 group compared with the other highland barley intervention groups (*p* < 0.05).

**Figure 6:**
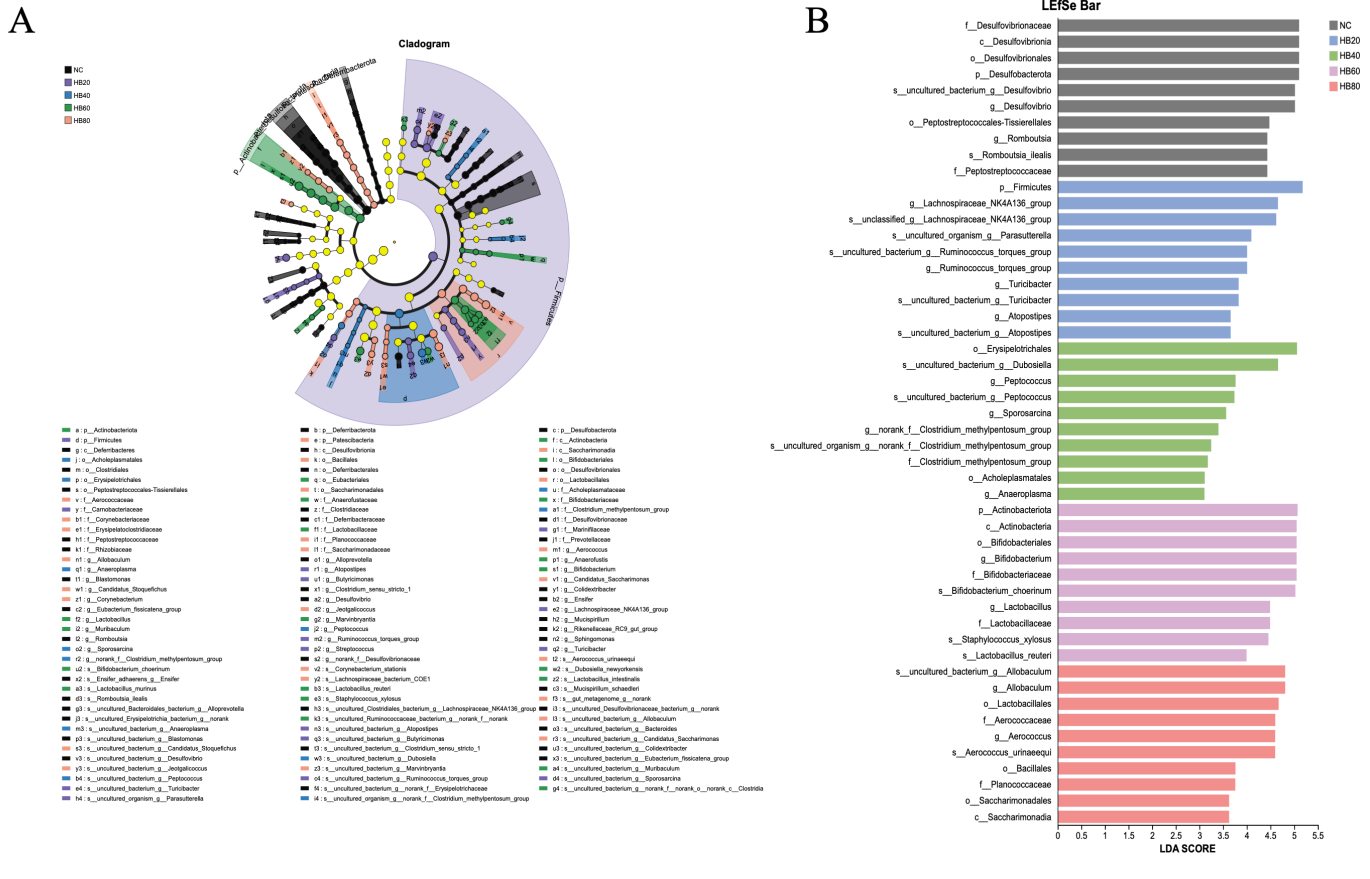
(**A**) taxonomic cladogram showing the relative abundance of gut microbiota across all groups, with circles representing phylogenetic levels from phylum (inner circle) to species (outer circle), and circle diameter proportional to the relative abundance of each taxon; (**B**) linear discriminant analysis effect size (LEfSe) comparison of gut microbiota among groups with an LDA score > 3.

To systematically identify microbial taxa with discriminative significance among dietary groups, LEfSe analysis was further performed (Figure 6A,B). The phylogenetic cladogram revealed enrichment of distinct taxa at multiple taxonomic levels across different highland barley supplementation groups (Figure 6A). LDA effect size analysis identified characteristic taxa with significant discriminative power (LDA score > 3) in each group, further confirming that highland barley dietary intervention substantially reshaped gut microbiota structure at the genus and higher taxonomic levels (Figure 6B). In the control group (NC), *Desulfovibrio* and its related taxa (e.g., *Desulfovibrionaceae* and *Desulfobacterota*) exhibited high LDA scores, indicating enrichment of sulfate-reducing–associated taxa. In the HB20 group, *Lachnospiraceae_NK4A136_group* and *Ruminococcus_torques_group* were significantly enriched and served as representative discriminative taxa. In the HB40 group, genera such as *Dubosiella* and *Peptococcus* displayed higher LDA scores. In the HB60 group, *Bifidobacterium* and *Lactobacillus*–related taxa (e.g., *Bifidobacteriaceae* and *Lactobacillaceae*) were significantly enriched and constituted the major discriminative taxa. In the HB80 group, *Aerococcus* and *Allobaculum* exhibited high discriminative effect values, indicating the formation of a distinct genus-level microbial profile.

### 3.6. Associations Between Gut Microbiota and Hypoxia-Related Physiological Parameters

**Figure 7:**
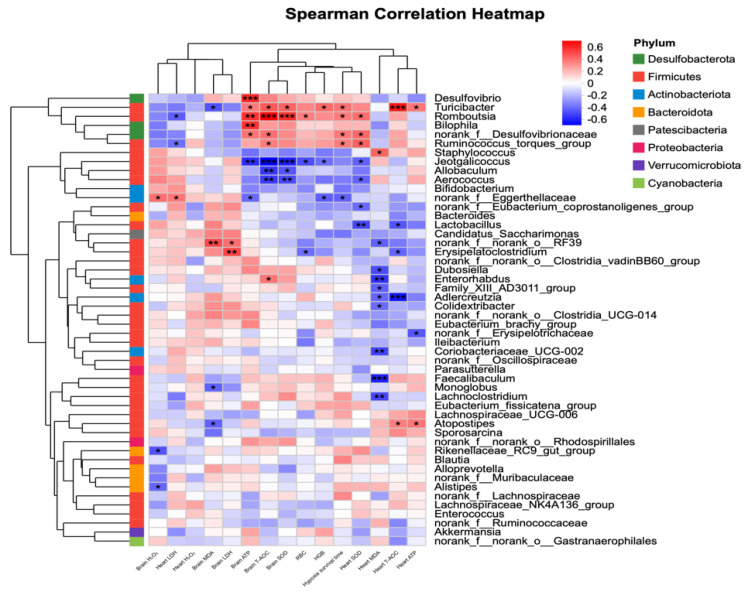
Spearman correlation analysis between genus-level gut microbiota and hypoxia-related physiological parameters. Heatmap showing Spearman correlation coefficients between the relative abundance of the top 50 genera and hypoxia survival time, hematological indices, oxidative stress markers, and energy metabolism–related parameters. Unclassified taxa were excluded prior to analysis. Colors indicate the strength and direction of correlations (red, positive; blue, negative). Statistical significance is indicated by *p* < 0.05 (*), *p* < 0.01 (**), and *p* < 0.001 (***). Hierarchical clustering was performed using average linkage.

Spearman correlation analysis showed that the relative abundances of several gut genera were significantly associated with hypoxic survival time and key physiological parameters (Figure 7). Specifically, *norank_f__Eggerthellaceae* was significantly negatively correlated with hypoxic survival time (ρ = −0.402, 0.01 < *p* ≤ 0.05), whereas *Ruminococcus_torques_group* (ρ = 0.367, 0.01 < *p* ≤ 0.05), *norank_f__Desulfovibrionaceae* (ρ = 0.381, 0.01 < *p* ≤ 0.05), *Turicibacter* (ρ = 0.395, 0.01 < *p* ≤ 0.05), and *Romboutsia* (ρ = 0.373, 0.01 < *p* ≤ 0.05) were significantly positively correlated with hypoxic survival time. In addition, regarding energy metabolism–related parameters, the relative abundance of *Desulfovibrio* was significantly positively correlated with ATP levels in brain tissue (ρ = 0.594, *p* ≤ 0.001). With respect to antioxidant indices, the relative abundance of *Turicibacter* was significantly positively correlated with T-AOC levels in heart tissue (ρ = 0.637, *p* ≤ 0.001).

Overall, these correlation results were consistent with the distribution patterns of microbial abundance and the observed phenotypic trends, providing supportive evidence for potential associations between gut microbiota alterations and hypoxia-related physiological responses.

## 4. Discussion

In the present study, a normobaric acute asphyxial hypoxia model was employed to systematically evaluate the effects of different inclusion levels of highland barley on hypoxia tolerance in mice. The results showed that dietary supplementation with highland barley did not markedly affect body weight or food intake across the tested inclusion levels. Under these conditions, mice in the HB20 group exhibited a longer hypoxia survival time than those in the basal diet control group (61.04 ± 2.39 min vs. 51.56 ± 3.52 min), suggesting that moderate highland barley intake may be associated with a favorable phenotypic response. It should be noted that the estimated difference between the HB20 and NC groups was accompanied by a relatively wide 95% confidence interval, indicating a degree of uncertainty in the magnitude of the effect. Accordingly, this difference is more appropriately interpreted as a positive trend. Overall, hypoxia survival time displayed a non-linear dose-dependent pattern, and higher inclusion levels (HB60–HB80) did not confer additional benefits, indicating the presence of a relatively appropriate intake range for highland barley-based dietary intervention.

### 4.1. Oxidative Stress–Related Basis for the Improvement of Hypoxia Tolerance by Moderate Highland Barley Intake

Hypoxia can disrupt mitochondrial electron transport and promote reactive oxygen species (ROS) accumulation, thereby challenging redox homeostasis, particularly in high-oxygen-demand organs such as the brain and heart [21,22]. Hydrogen peroxide (H_2_O_2_) is often used as an indicator of oxidative burden because of its relative stability and diffusibility [23,24]. Endogenous antioxidant defenses (e.g., SOD and total antioxidant capacity, T-AOC) counterbalance ROS, whereas malondialdehyde (MDA) reflects lipid peroxidation–associated damage [25,26,27]. In this study, the HB20 diet was associated with lower H_2_O_2_ and MDA levels and higher SOD activity and T-AOC in both brain and heart tissues under normobaric asphyxial hypoxic challenge. Together, these changes indicate an improved redox profile and reduced lipid peroxidation, which provides a plausible physiological basis for the favorable hypoxia-tolerance phenotype observed at moderate inclusion levels. Similar antioxidant-oriented responses to cereal-derived bioactives, including polyphenols and polysaccharides, have been reported in hypoxia-related models [28,29].

### 4.2. Role of Energy Metabolic Homeostasis in Hypoxia Tolerance

In addition to oxidative stress, disruption of energy metabolism represents a critical determinant of hypoxia tolerance and survival outcomes under acute hypoxic conditions. Insufficient oxygen availability directly suppresses mitochondrial oxidative phosphorylation, leading to reduced ATP production [30]. In parallel, LDH, a key enzyme involved in glycolysis-related pathways, is commonly used to reflect the intensity of metabolic responses and the risk of tissue injury under hypoxic conditions [27].

In the present study, under normobaric acute asphyxial hypoxia, ATP levels in the brain tissue of the HB20 group were increased and accompanied by a significant reduction in LDH activity. Similar trends were observed in cardiac tissue, consistent with previous findings reported by Cai et al. [9]. These results suggest that moderate highland barley supplementation may help maintain energy supply capacity under hypoxic stress while reducing reliance on anaerobic metabolism, thereby alleviating metabolic burden in key organs during acute hypoxia [31]. Given that ATP availability is closely associated with membrane integrity, ion transport, and the maintenance of essential cellular functions, the observed improvement in energy metabolic status provides a plausible physiological explanation for the prolonged hypoxia survival time observed in the HB20 group, in agreement with the findings of Ma et al. [32].

Notably, with further increases in highland barley inclusion levels (HB60–HB80), energy metabolism-related indicators did not show sustained improvement, indicating that the supportive effects of highland barley on hypoxic energy homeostasis are likewise confined to an appropriate dosage range [12]. This finding is consistent with the nonlinear dose–response pattern observed for survival time and further emphasizes that, in nutritional interventions targeting acute hypoxia, appropriate intake levels may confer greater physiological benefits than higher doses.

### 4.3. Changes in Hematological Parameters and Gut Microbiota

At the systemic level, hypoxia tolerance is shaped not only by oxidative stress and energy metabolism in specific organs but also by circulating oxygen transport capacity and the overall metabolic environment [33]. It should be noted that hematological parameters in the present study were measured immediately after completion of the normobaric acute asphyxial hypoxia challenge; therefore, the terminal sampling time point may have influenced the absolute values of certain indicators. Accordingly, the present analysis primarily focuses on relative differences and dose–response trends among dietary groups. RBC [34] and HGB [35] reflect basal oxygen-carrying and oxygen-delivery capacity, and previous studies have shown that hypoxic stimulation can induce hematopoietic adaptations to enhance oxygen transport. In this study, dietary supplementation with 20% highland barley moderately increased red blood cell counts and hemoglobin levels under hypoxic conditions, which may enhance blood oxygen-carrying capacity and support tolerance to acute hypoxia, consistent with findings reported by Cui et al. [36]. These changes align with hypoxia-compensatory physiological mechanisms, whereby enhanced erythropoiesis improves oxygen supply and utilization to support aerobic metabolism [37]. However, this effect did not progressively increase with higher levels of highland barley supplementation, indicating that hematological regulation alone is unlikely to fully account for the observed differences in hypoxia tolerance phenotypes. When considered together with the regulation of oxidative stress and energy metabolic homeostasis, these results suggest that multiple physiological processes jointly contribute to the formation of hypoxia tolerance [9].

As an important systemic regulatory factor, the gut microbiota may participate in stress adaptation by modulating energy substrate utilization, redox homeostasis, and mucosal immune barrier function [38]. In the present study, 16S rRNA sequencing of cecal contents under normobaric acute hypoxia demonstrated that highland barley dietary intervention induced dose-dependent remodeling of the gut microbiota. In terms of α-diversity, both the Simpson and Chao indices were significantly increased in the HB20 and HB40 groups compared with the NC group, indicating enhanced microbial richness and evenness within this intake range. In contrast, no further increases were observed in the HB60 and HB80 groups, and the Simpson index exhibited a declining trend, suggesting that higher inclusion levels did not confer sustained diversity benefits [12]. β-diversity analyses (PCoA and NMDS) further revealed clear separation among dietary groups, indicating substantial alterations in overall microbial community structure in response to different levels of highland barley intake [39]. At the phylum level, Firmicutes, Actinobacteriota, and Bacteroidota were the dominant taxa across groups, with significant shifts in their relative abundances, suggesting that dietary intervention broadly reshaped the gut microbial ecosystem under hypoxic conditions.

Further genus-level analyses revealed that highland barley intervention was accompanied by differential shifts in multiple genera associated with metabolic regulation and intestinal barrier homeostasis, providing supportive evidence for its involvement in the systemic modulation of host hypoxic status. In the hypoxia control group (NC), a relatively high abundance of *Desulfovibrio* and its associated taxa was observed, consistent with previous findings reported by Yang et al. in hypoxic models [39]. *Desulfovibrio*, a representative sulfate-reducing bacterium, has been widely reported to predominate under physiological conditions characterized by impaired intestinal barrier function and increased inflammatory burden [40]. In the present study, highland barley intervention was associated with an overall reduction in the relative abundance of this taxon (Figure 5C), suggesting a potential role in alleviating unfavorable gut microbial features under hypoxic conditions. In contrast, *Lachnospiraceae_NK4A136_group* has been linked to intestinal barrier repair and the maintenance of microbiota homeostasis and is considered highly responsive to changes in the gut microenvironment [41]. Consistently, this genus exhibited discriminatory enrichment in the HB20 group (Figure 6B), indicating that moderate barley supplementation may contribute to the regulation of energy substrate utilization and adaptive modulation of the mucosal microenvironment under hypoxic conditions through the remodeling of anaerobic fermentation-associated gut metabolic networks [12]. Moreover, *Bifidobacterium* displayed a non-dose-dependent abundance pattern across different barley supplementation levels (Figure 5A). This genus is known for its strong anaerobic adaptability and its involvement in host responses to adverse environments through mechanisms including the promotion of short-chain fatty acid production, enhancement of intestinal barrier function, and modulation of host oxidative stress [9,42]. The observed changes in Bifidobacterium under barley intervention suggest that gut microbial modulation may provide indirect support for host tolerance to hypoxic stress by improving the local metabolic environment [43].

Previous studies have demonstrated that gut-derived short-chain fatty acids (SCFAs), particularly acetate, can be absorbed from the intestine and enter the systemic circulation [44]. Concurrently, the blood–brain barrier expresses multiple monocarboxylate transport systems, including monocarboxylate transporters (MCTs), which facilitate the translocation of monocarboxylate metabolites such as acetate, lactate, and ketone bodies across the barrier [45]. Together, these features provide a physiological basis for the potential involvement of gut-derived SCFAs in the regulation of brain energy metabolism during acute stress conditions [46]. In addition, emerging evidence indicates that SCFAs may participate in the maintenance of brain ATP homeostasis through the modulation of energy-sensing signaling pathways, including the mammalian target of rapamycin (mTOR) pathway [47], and are closely associated with brain physiological function and behavioral performance [48,49]. It should be emphasized that blood–brain barrier permeability was not directly assessed in the present study; therefore, the proposed links remain largely associative and warrant further validation through targeted metabolomic analyses and direct assessment of blood–brain barrier–related parameters in future studies.

### 4.4. Appropriate Dosage and Its Potential Nutritional Significance

Across outcomes, the response to dietary highland barley was non-linear: improvements were more evident in the 20–40% range, whereas higher inclusion levels did not confer additional benefit. This overall pattern was consistent with survival time, indices of oxidative status, energy metabolism, and gut microbiota measures (Figure 1, Figure 2, Figure 3, Figure 4 and Figure 5). Highland barley provides dietary fiber, β-glucan, polyphenols, and polysaccharides, which at appropriate intake levels may support antioxidant defense, energy homeostasis, and gut microbial modulation under stress conditions [11]. However, increasing whole-grain inclusion to high proportions can substantially reshape the dietary matrix, with potential consequences for nutrient bioavailability and the profile of fermentable substrates available to the gut microbiota [50,51,52].

It should be further noted that at higher dietary highland barley inclusion levels (e.g., HB60–HB80), even with an isocaloric formulation, the relative dietary supply of certain micronutrients and antinutritional factors may change substantially. According to estimates in Supplementary Table S3, increasing dietary highland barley from 20% to 60% and 80% raised the dietary supplies of dietary fiber, β-glucan, and crude polysaccharides to 61.38–81.84, 14.71–19.61, and 67.25–89.67 g/kg diet, respectively. In this context, given that highland barley is used as a whole-grain ingredient, the absolute intake of antinutritional components such as phytic acid may also increase. Higher levels of phytic acid have been reported to decrease the bioavailability of minerals (e.g., iron and zinc) via chelation, which could indirectly influence enzyme systems related to energy metabolism and antioxidant defense under certain conditions [53]. Therefore, the lack of a progressive enhancement in antihypoxic outcomes observed in the high-inclusion groups may reflect the combined influence of shifts in overall dietary structure and potential cumulative effects of antinutritional factors [54]. Taken together, these observations are consistent with a dietary matrix–dependent, non-linear dose–response pattern, in which higher inclusion levels may alter nutrient bioaccessibility and fermentable substrate availability and thereby attenuate physiological responsiveness.

### 4.5. Study Limitations and Future Perspectives

Several limitations of the present study should be considered. First, the sample size in each group was *n* = 8. This design was informed by sample sizes commonly used in previous normobaric asphyxial hypoxia animal studies and is adequate for capturing major physiological responses to hypoxic stress. However, the limited sample size may reduce statistical power for detecting moderate effect sizes or subtle differences between adjacent supplementation levels. To enhance transparency and facilitate interpretation, effect sizes and their 95% confidence intervals were additionally reported for key outcomes, particularly hypoxic survival time. Second, no formal blinding procedure was implemented. All experimental procedures were conducted under standardized conditions, and outcomes were primarily derived from relative comparisons among dietary groups tested under identical experimental settings. In addition, oxidative stress–, energy metabolism–, and hematological–related parameters were assessed at the terminal time point following the acute normobaric asphyxial hypoxia survival test. As terminal hypoxic exposure represents a strong acute stressor, it may have influenced certain physiological measurements. Accordingly, the reported results mainly reflect relative differences and dose–response trends among dietary groups under the same terminal hypoxic stress, rather than basal resting states after long-term dietary intervention. Hematological indices may also be affected by terminal stress-related factors, such as hemoconcentration or hemolysis. Finally, gut microbiota analysis in this study was based on an OTU clustering pipeline using a 97% sequence similarity threshold to ensure comparability with previous literature. Future studies will prioritize ASV-based analytical approaches to further improve taxonomic resolution and to validate the microbial features observed in this study, as well as their potential functional implications.

## 5. Conclusions

This study systematically evaluated the effects of dietary highland barley supplementation at different inclusion levels on tolerance to normobaric acute hypoxia in mice. Overall, a moderate inclusion level (20%) was associated with a longer hypoxic survival time, whereas higher inclusion levels did not confer additional benefits, suggesting a non-linear dose–response pattern and a potentially effective intake range for hypoxia-related nutritional interventions. Across key physiological readouts, the favorable profile observed at moderate inclusion was accompanied by improved oxidative stress-related indices and a more favorable energy metabolism status in brain and heart tissues, including higher antioxidant defense capacity, lower lipid peroxidation markers, and better preservation of ATP availability under the terminal hypoxic challenge. In parallel, highland barley supplementation altered gut microbiota composition, and moderate inclusion levels were associated with increased microbial diversity and shifts in multiple genera linked to metabolic homeostasis and intestinal barrier function.

Taken together, these findings indicate that the antihypoxic potential of highland barley is not simply dependent on higher intake levels but is more consistent with an appropriate dietary proportion. This work provides experimental evidence supporting the rational use of highland barley in hypoxia-related nutritional strategies. Nevertheless, this study did not isolate the contributions of individual barley components, and microbiota–phenotype relationships remained associative. Future studies using parallel baseline cohorts, targeted component interventions, and mechanistic approaches are warranted to clarify causal pathways and to refine the effective intake range.

## Data Availability

The data presented in this study are available on request from the corresponding author.

## Supplementary Materials

The following supporting information can be downloaded at: https://www.mdpi.com/article/10.3390/nu18040659/s1, Figure S1: Gut microbiota community composition at the phylum and genus levels in mice following dietary highland barley intervention; Table S1: Energy ratio provided by nutrients (%); Table S2: Composition of experimental diets; Table S3: Estimated amounts of selected highland barley–derived components across experimental diets with different inclusion levels.
