# Supplementary material for: Effects of Dietary Highland Barley at Different Inclusion Levels on Normobaric Hypoxia Tolerance, Oxidative Stress, Energy Metabolism, and Gut Microbiota in Mice
Source: Nutrients. 2026 Feb 17;18(4):659. doi: 10.3390/nu18040659 (PMC12942744; doi:10.3390/nu18040659)
Supplement: Supplementary file 1 [file nutrients-18-00659-s001.zip › nutrients-4131089-supplementary.pdf]

Table S1 Energy ratio provided by nutrients（%）

Ingredient	Control Group （NC）	Intervention Group			
		HB20	HB40	HB60	HB80
Protein	20	20	20	20	20
Carbohydrate	70	20	20	20	20
Fat	10	60	60	60	60
Total			100		

Table S2 Composition of experimental diets

	NC		HB20		HB40		HB60		HB80	
	g%	kcal%	g%	kcal%	g%	kcal%	g%	kcal%	g%	kcal%
Qingke	0	0	230.15	753.83	460.25	1507.48	690.46	2261.48	920.61	3015.29
Casein, 30 Mesh	140	560	110.77	443.08	81.54	326.16	52.3	209.2	23.07	92.28
L-Cystine	1.8	7.2	1.8	7.2	1.80	7.20	1.8	7.2	1.80	7.20
Corn Starch	495.69	1983	352.52	1410.09	209.38	837.53	66.182	264.728	0.00	0.00
Maltodextrin 10	125	500	125	500	125.00	500.00	125	500	48.01	192.05
Sucrose	100	400	100	400	100.00	400.00	100	400	100.00	400.00
Cellulose	50	0	29.54	0	9.08	0.00	0	0	0.00	0.00
Soybean Oil	40	360	32.86	295.74	25.73	231.57	18.59	167.31	11.46	103.14
t-Butylhydroquinone	0.01	0	0.01	0	0.01	0.00	0.008	0	0.01	0.00
Mineral Mix S10022M	35	0	35	0	35.00	0.00	35	0	35.00	0.00
Vitamin Mix V10037	10	40	10	40	10.00	40.00	10	40	10.00	40.00
Choline Bitartrate	2.5	0	2.5	0	2.50	0.00	2.5	0	2.50	0.00
Total	1 000.00	3850.2	1 030.15	3849.94	1060.29	3849.94	1101.84	3849.918	1152.46	3849.96

Table S3 Estimated amounts of selected highland barley–derived components across experimental diets with different inclusion levels

Component	HB20 (g/kg diet)	HB40 (g/kg diet)	HB60 (g/kg diet)	HB80 (g/kg diet)
Dietary fiber	20.46	40.92	61.38	81.84
β-glucan	4.9	9.8	14.71	19.61
Polyphenols	0.76	1.52	2.28	3.04
Crude polysaccharides	22.42	44.83	67.25	89.67

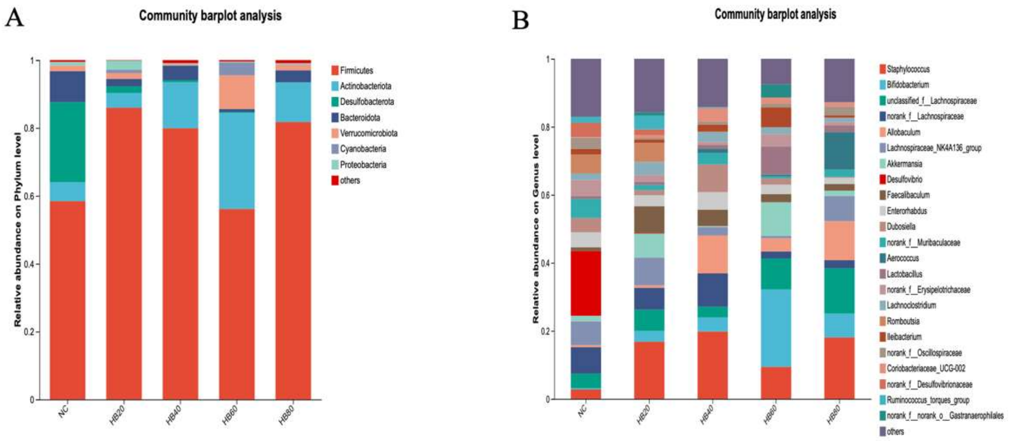

Figure S1. Gut microbiota community composition at the phylum and genus levels in mice following dietary highland barley intervention. (A) Relative abundance of gut microbiota at the phylum level; (B) Relative abundance of gut microbiota at the genus level.

Relative abundances were calculated based on 16S rRNA gene sequencing data. Each bar represents the mean community composition within a dietary group.
